# Analysis and comparison of electron radiation damage assessments in Cryo-EM by single particle analysis and micro-crystal electron diffraction

**DOI:** 10.3389/fmolb.2022.988928

**Published:** 2022-10-05

**Authors:** Dan Shi, Rick Huang

**Affiliations:** ^1^ Center for Structural Biology, Center for Cancer Research, National Cancer Institute, Frederick, MD, United States; ^2^ Laboratory of Cell Biology, Center for Cancer Research, National Cancer Institute, Bethesda, MD, United States

**Keywords:** cryo-EM, radiation damage assessment, site-specific and global damages, SPA, MicroED

## Abstract

Electron radiation damage to macromolecules is an inevitable resolution limit factor in all major structural determination applications using cryo-electron microscopy (cryo-EM). Single particle analysis (SPA) and micro-crystal electron diffraction (MicroED) have been employed to assess radiation damage with a variety of protein complexes. Although radiation induced sidechain density loss and resolution decay were observed by both methods, the minimum dose of electron irradiation reducing high-resolution limit reported by SPA is more than ten folds higher than measured by MicroED using the conventional dose concept, and there is a gap between the attained resolutions assessed by these two methods. We compared and analyzed these two approaches side-by-side in detail from several aspects to identify some crucial determinants and to explain this discrepancy. Probability of a high energy electron being inelastically scattered by a macromolecule is proportional to number of layers of the molecules in its transmission path. As a result, the same electron dose could induce much more site-specific damage to macromolecules in 3D protein crystal than single particle samples. Major differences in data collection and processing scheme are the key factors to different levels of sensitivity to radiation damage at high resolution between the two methods. High resolution electron diffraction in MicroED dataset is very sensitive to global damage to 3D protein crystals with low dose accumulation, and its intensity attenuation rates at atomic resolution shell could be applied for estimating ratio of damaged and total selected single particles for SPA. More in-depth systematically radiation damage assessments using SPA and MicroED will benefit all applications of cryo-EM, especially cellular structure analysis by tomography.

## Introduction

When cryo-EM emerged as a powerful tool in structural biology over four decades ago, electron radiation damage to protein crystals was first identified as a major resolution limit factor for high resolution structure determination [Glaeser, 1971; Unwin and Henderson 1975]. Because impact from electron beams and thermal induced motion significantly exceed radiation damage for cryo-EM single particle analysis (SPA) in the early days [Li et al., 2013 and Grigorieff 2013], electron crystallography was the primary method to obtain high resolution structure information from 2D membrane and thin 3D protein crystals [Taylor and Glaeser, 1976; Henderson et al., 1986; Wang and Kuhlbrandt, 1992; Shi et al., 1995]. Intensity decay in high-resolution diffraction shells in series of exposures were employed for measuring radiation damage and resolution reduction as electron dose accumulates [Chiu et al., 1981, Jeng and Chiu, 1984, Stark et al., 1996, Baker et al., 2010, and Bammes et al., 2010].

In the last decade, many novel technologies and methodologies have been developed and implemented in SPA, in which the direct electron CMOS detector is the major key breakthrough invention [Milazzo et al., 2011 and Bammes et al., 2012]. Its high frame read-out feature enables image processing with dose fractionated movies to correct beam and thermal induced motions, which unprecedently improves image quality [Grigorieff 2013 and Li et al., 2013]. Solving de novel macromolecular structures at atomic resolution has become a routine pipeline for SPA.

The other new powerful tool developed in parallel to SPA is sub-micrometer 3D crystal electron diffraction (MicroED) [Shi et al., 2013] as a part of 3D electron crystallography [Shi et al., 1998 and Zhang et al., 2010] that allows 3D protein crystal structures to be determined at atomic resolution by using extremely low electron dose [Nannenga et al., 2014a; Rodriguez et al., 2015]. After beam-induced motion becomes correctable, radiation damage in macromolecules has become the primary outstanding resolution limit factor in all cryo-EM applications. Recently, SPA and MicroED were employed to assess radiation damage at high resolution. Both methods exhibited similar side chain density degeneration caused by inelastic electron scattering. However, there is a significant discrepancy in rate and severity of resolution decay between the two methods. Full understanding of SPA and MicroED in data acquisition and analysis will facilitate radiation damage assessment and provide solution to minimize such effect for all cryo-EM applications.

## Cryo-EM data acquisition and radiation damage

**FIGURE 1:**
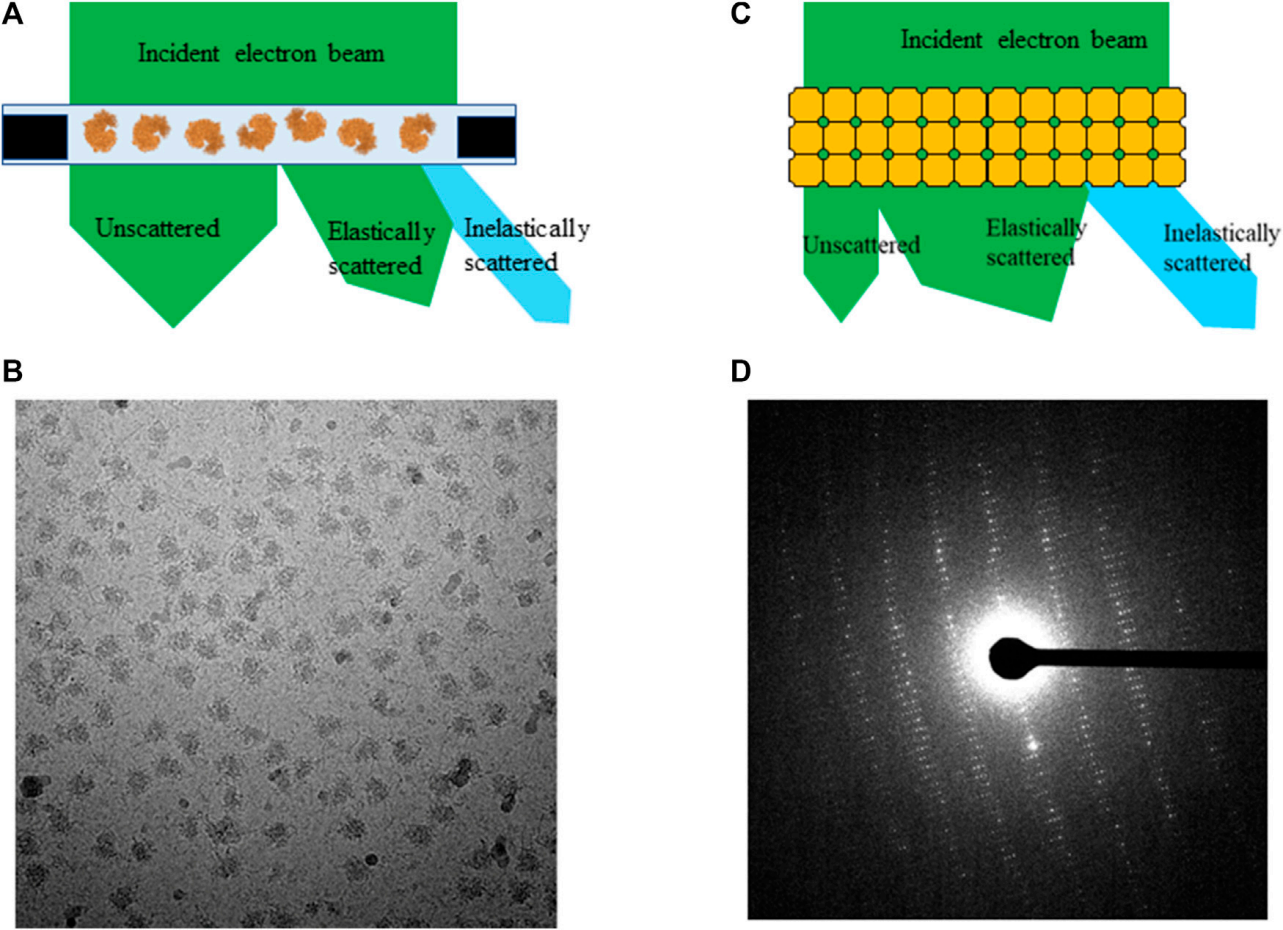
An electron beam transmits through **(A)** frozen-hydrated single particle sample and **(C)** 3D protein crystal, **(B)** projection image of single particles and **(D)** electron diffraction pattern from proteinase K crystal.

When electrons penetrate through a thin frozen-hydrated biological sample, three types of outgoing electrons reach the detector: unscattered which electrons do not make any atomic interaction, elastically and inelastically scattered by atoms in a sample during transmission. Because of strong interaction between electrons and specimen, the ratio among these three types of outgoing electrons greatly depends on sample dimensions and its elemental composition. Vast majority sizes of macromolecules and complexes in near biological-native buffer are under 50 nm, which is much shorter than electron inelastic mean free path (200–300 nm) in frozen-hydrated biological sample [Malis et al., 1988 and Grimm et al., 1996 and Martynowycz et al., 2021]. Elastically scattered electrons shift their phases that correspond to their interactions with different atoms comparing to no phase shift for unscattered electrons, and large angle scattering may be cut out by an objective aperture resulting an increase in amplitude contrast. Unscattered and elastically scattered electrons carry out macromolecule structure signals to form weak phase contrast projection image of single particles as shown in Figures 1A,B.

By contrast, electron diffraction patterns (EDP) are formed by interference of electrons being elastically scattered at back focal plane of objective lens while unscattered electrons are focused on the center of a diffraction pattern called direct beam, as shown in Figures 1C,D. The intensity of direct beam is reverse proportional to sample thickness as a function of electron inelastic mean free path for 3D protein crystals and other thick biological samples [Martynowycz et al., 2021]. Inelastic scattering electrons deposit their kinetic energy onto sample as forms of radiation damages, e.g., ionization, breaking covalent bonds, and atom delocalization as total dose accumulates throughout data acquisition in all applications of cryo-EM. Any inelastically scattered electrons, whose wavelength and phase are changed, would produce white noise when captured by the detector in both bright field and diffraction images unless removed by prisms and lens sets in an energy filter.

## Radiation damage assessment from SPA

A typical high quality frozen-hydrated single particle sample contains hundreds of thousands of macromolecules embedded in a very thin layer of vitreous ice illustrated in Figure 1A. An electron microscope equipped with a direct electron detector records projection images of individual macromolecules in a format of dose fractionated movies at a total dose ranging from 30 e^−^/Å^2^ to 60 e^−^/Å^2^. Beam induced motion is corrected by aligning and summing subframes prior to data processing, and noises from radiation damage remain in data as one of the unresolved outstanding resolution limit factors. Each micrograph is a summation of macromolecules with damage progressing from low to high. Few macromolecular complexes were hired for assessing radiation damage with accumulated total dose up to 100 e^−^/Å^2^. They are chosen for dose assessment experiments due to their high homogeneity (>95%) and X-ray structures at atomic resolution are available for direct comparison [Roberts and Davies, 2012]. Therefore, the entire selected particle stacks were used for 3D reconstruction at different dose accumulations. When only a small subgroup of frames with subtotal dose under 3 e^−^/Å^2^ were summed, the attained resolutions were significantly reduced [Grant and Grigorieff 2015] because signals in such low dose frames are insufficient for generating high resolution reconstruction.

**FIGURE 2:**
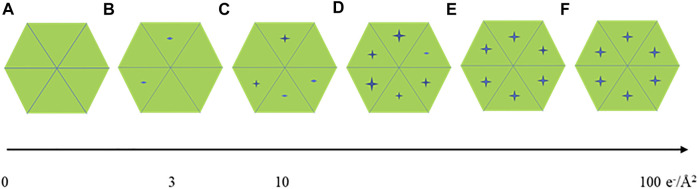
A cartoon illustration of a hexameric complex being gradually damaged by electron dose accumulation from **(A–F)**.

For seeking the optimal exposure time, summation of different subsets of frames starting from 10 e^−^/Å^2^ were used for evaluating radiation damage. Their statistical data are summarized in Table 1 along with the radiation damage assessment from MicroED, which shows the estimated optimal doses for high resolution data collection are quite different between these two methods. Gold-standard Fourier shell correlation (FSC) was used as a quantitative resolution readout to evaluate the outcome of radiation damage [Grant and Grigorieff 2015], and electron density losses were observed by 3D reconstruction at higher total dose accumulations [Bartesaghi et al., 2014]. Figures 2A–F illustrate how radiation damage to a hexametric complex could gradually build up as electron dose accumulates. When only low dose, such as less than 10 e^−^/Å^2^, were summed for reconstruction, not every subunit in the complexes was damaged as shown in Figures 2A–C. Reconstructions were calculated from projection images of complexes shown in Figure 2A, 2a+2b and/or figure 2a+2b+2c, in which undamaged subunits would contribute most of signals in the electron density. As more consecutive frames in a dose fractionated movie were summed for data processing, frames recorded from later dose series (Figures 2D–F) would be combined with the early dose (undamaged) series (Figures 2A–C), and the signal to noise (SNR) ratio would progressively increase at undamaged low resolution backbone portion and diminish at those damaged regions. Therefore, global resolution measured by FSC would be reduced as well as electron density loss at the radiation damage region becomes more visible.

**TABLE 1:** Radiation damage assessment using SPA and MicroED with biological complexes. Resolution limit is determined by global gold-standard FSC at 0.145 for SPA and measurable diffraction spots for MicroED, respectively.

	β-galactosidase^a^	Rotavirus VP6^b^	20s Proteasome^b^ ^,^ ^c^	Proteinase K^d^	Hepta-peptide^d^
Method	SPA	SPA	SPA	MicroED	MicroED
Symmetry	D2	I, T = 13	D7	D4	C1
Subunits number	4	780	28	>10^6^*	>10^6^*
Particles number	23,452	4,187	49,954	<10**	<10**
Optimal dose e^−^/Å^2^	10	16.1	12.6	0.9	0.27
Resolution Å	3.2	2.6	2.8	1.7	1.01
Maximum dose e^−^/Å^2^	50	100	53	8	3.76
Resolution Å	N/A	2.9	3.0	3.2	1.4
Site-specific damage	acidic side chains	acidic side chains		acidic side chains, disulfide bonds	Zn atom, side chains

a – Bartesaghi et al., 2014.

b – Grant and Grigorieff 2015.

c – Campbell et al., 2015.

d – Hattne et al., 2018.

*number of unit cells in a crystal.

**number of crystals.

## Radiation damage measured by MicroED

**FIGURE 3:**
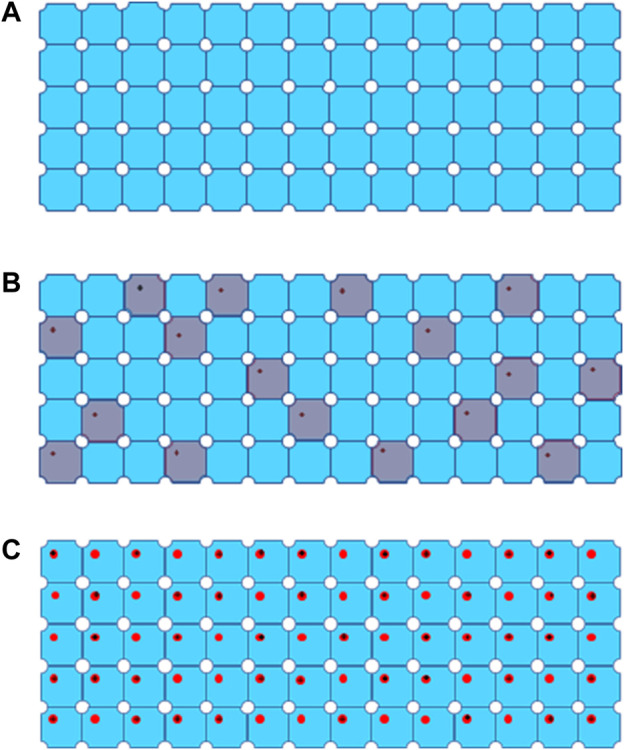
Process of global damage to a 3D protein crystal from **(A)** a well-ordered crystal without radiation damage, **(B)** global damage after extremely low dose irradiation, defected unit cells are colored in grey, to **(C)** global damage with progressively high dose marked by black cross for site specific damage regions.

In 3D electron crystallography, electron beam is usually widely spread to illuminate the entire or majority area of a crystal, which is composed of more than a million of macromolecules periodically packed in 3D space, and it could be viewed as a huge complex with translational symmetry, as shown in Figure 3A. Electron diffraction patterns (EDP) record 3D high resolution structure information in Fourier (reciprocal) space. Each spot in EDP contains structure signals simultaneously averaged from all macromolecules in a crystal at a given crystalline plane or spatial frequency. This feature enables acquisition of high resolution diffraction data at low dose rate under 0.01 e^−^/Å^2^/s [Shi et al., 1998]. The intensity I_0_ for diffraction spot [H,K,L] from a well ordered or perfect 3D crystal can be expressed:

$$I_{0}\left(HKL\right)=\underset{Vc}{\int }\rho \left(r\right)e^{-i2\pi R\cdot r}dr$$

$$\propto {Ν}_{\text{a}}^{2}{Ν}_{\text{b}}^{2}{Ν}_{\text{c}}^{2}{\left|\underset{v}{\int }\rho \left(r\right)e^{-i2\pi \left(\frac{xH}{a}+\frac{yK}{b}+\frac{zL}{c}\right)}dr\right|}^{2}=N_{0}^{2}{\left|e^{-\varphi \left(H,Κ,L\right)}\right|}^{2}$$ (1)

V_c_ is whole crystal volume illuminated by electron beam, **R** and **r** are vectors in reciprocal space and real space respectively, N_a_, N_b_ and N_c_ are number of unit cells along three crystal axis, N_0_ = N_a_N_b_N_c_, the integration for structure factor is through the whole unit cell volume v , and ρ(***r***) is electron density. The diffraction intensity is directly proportioned to both phases and square of repeating units in all three dimensions.

Data collection scheme of MicroED was described in previous publications [Shi et al., 2016, Hattne et al., 2018], in which a dataset consists of series discrete diffraction patterns consecutively recorded from a continuously rotating crystal, at 0.05 e^−^/Å^2^ or lower dose per frame. Diffraction intensities decay in different resolution shells were sampled and plotted as higher dose progressively accumulates. Site specific damage to individual macromolecules was visualized using dataset with higher accumulated dose in 3D reconstruction [Hattne et al., 2018 and Nannenga et al., 2018], and their results are summarized in Table 1 in comparison to radiation damage assessed by SPA. When an electron beam transmits through a 3D crystal, inelastic scatterings would randomly disperse in unit cells along the electron beam path to induce site specific damage to macromolecules. What an EDP decay records is global damage to a crystal comprised of all the unit cells with site-specific damage, as shown in Figures 3B,C as electron dose accumulates. If a site-specific damaged region is relatively small comparing to the whole macromolecule, then it only appears as a minor defect in a unit cell with the majority of macromolecules remain undamaged in a crystalline structure [Hattne et al., 2018]. Any ionization or bond breaking could have a major impact on structure factor ϕ for each individual defected unit cell.

The integration through the whole crystal in expression would become very complicated, because the structure factors in undamaged unit cells would remain as in expression (1) and structure factors or phases would vary in different defected unit cells. For simplifying calculation of diffraction intensity, we use two separated approaches for estimating diffraction intensity decay in high resolution shell and low resolution shells, respectively.

High resolution spots are generated from large angle scattering by atoms on crystalline planes in a particular spacing to meet Bragg’s condition/law. Therefore, the phases are sensitive to any small local change caused by ionization and delocalization. The integration of whole crystal in expression (1) can be separated into two parts, i.e., summations of every perfect unit cell and every defected unit cell shown in expression (2):

$$I_{d}\propto {\left({\text{N}}_{0}-{\text{N}}_{d}\right)}^{2}{\left|\underset{v}{\int }\rho \left(r\right)e^{-i2\pi \left(\frac{xH}{a}+\frac{yK}{b}+\frac{zL}{c}\right)}dr\right|}^{2}+\overset{N_{d}}{\underset{k}{\sum }}{\left|\underset{v}{\int }\rho_{k}\left(r\right)e^{-i2\pi R\cdot r}dr\right|}^{2}$$ (2)

*K* is through 1 to N_d_ which is the total number of defected unit cells shown in grey in Figure 3B. As a result, the second part of expression (2) may only generate background noise and the high resolution spots are mainly contributed by the first part of expression (2). Therefore, intensity decay in high resolution diffraction spots could be approximately estimated as:

$$I_{d}/I_{0}\propto {\left({\text{N}}_{0}-{\text{N}}_{d}\right)}^{2}/{\text{N}}_{0}^{2}$$ (3)

N_d_ is number of defected unit cells, as shown in Figure 3B. If N_d_ is over 70% of N_0_, average intensity of high-resolution diffraction spots will be dropped below 9% of the theoretical undamaged level, which might not be detectable even by highly sensitive CMOS detector [Nannenga et al., 2018].

For low resolution bins, the backbones of macromolecules can tolerate more electron damage and still remain structurally sound in original crystalline array [Grant and Grigorieff 2015 and Hattne et al., 2018]. So, the integration volume in expression (1) could be modified to v *’ =* v *-* v _*d*_ *,* v _*d*_ is total volume of site specific damage regions shown as red circles in Figure 3C, that could explain why intensity attenuation of low resolution spots were much less sensitive to electron dose. MicroED may not be able to accurately measure more severe radiation damage by very high electron dose irradiation.

## Discussion

Figures 1A,C illustrates an electron beam transmitting through a single particle specimen and a 3D crystal respectively. The obvious difference is that electron beam transmits only through a single layer of randomly orientated macromolecules in SPA specimen comparing to multiple layers of periodically packed macromolecules in 3D protein crystals. Since majority of single particle thickness is much less than electron inelastic scattering mean free path, large portion of electron beam may not interact with atoms in a single particle sample during exposure. In theory, when an electron travels through a 3D crystal (Figure 1C), the probability of being scattered inelastically should be at least multiple times higher than traveling through the same protein molecule in SPA specimen [Himes and Grigorieff 2021]. In other words, the same electron dose could induce multiple folds more site specific damages in a 3D crystal than the same or similar size proteins in a single particle sample. If crystal thickness is less than electron inelastic scattering mean free path, the amount of damaged macromolecules would be approximately proportional to number of layers in a crystal parallel to electron beam path. However, if the thickness is greater than the mean free path, impact from inelastic scattering and multiple scattering would be increased exponentially [Himes and Grigorieff 2021 and Martynowycz et al., 2021]. Specimen thickness should be considered as a critical factor for evaluating total global damage in 3D crystals.

The difference of data acquisition schemes for SPA and MicroED is another key factor for yielding a big gap in measured radiation tolerance. In a MicroED dataset, diffraction patterns can be used for plotting intensity attenuation in different resolution shells (highest resolution shell 1.01 Å) at 0.05 e^−^/Å^2^ or lower dose increment per frame [Hattne et al., 2018 and Nannenga et al., 2018]. Reflection intensity decay in a EDP is a direct and sensitive method to detect any global damage in a 3D crystal [Chiu et al., 1981]. In SPA, all signals were summed and averaged from undamaged transitioning to fully damaged state. SNR in undamaged low resolution region would progressively increase and gradually becomes weakened at high resolution as more damage regions and more frames with higher dose accumulation are included. The differential of SNR at undamaged and damaged regions could play a critical role for spotting site specific damage to macromolecules. Therefore, SPA may not be sensitive to localized or high resolution site specific damage, e.g., ionization and broken covalent bond, at very low dose accumulation until more frames recorded from more severe damaged particles at higher total dose are included to increase SNR differential between undamaged and damaged regions to a measurable level. Furthermore, SNR in high resolution shells could be elevated by averaging more particle images in 3D reconstruction. Using more homogeneous particles in SPA can improve attainable resolution from 3.2 Å and 2.6 Å to higher resolution as well as increase sensitivity and accuracy for radiation damage assessment [Bartesaghi et al., 2015]. Summation of frames in consecutive dose bins, e.g., 0–10, 11–20 e^−^/Å^2^ and so on, could more accurately assess radiation damage to macromolecules in order to determine rate and severity of damage progression in different accumulated doses. This radiation damage assessment data using this processing scheme have led to a dose weighing formula to be applied to all future datasets in SPA as well as cryo-ET [Grant and Grigorieff, 2015].

Global damage can also happen to protein complexes with higher symmetries, Figures 2A–F display the progress of a hexameric complex being gradually damaged during high dose movie acquisition. 2D classification may not be able to discern the difference among Figures 2A–C due to resolution limitation enforced computationally. Averaging a large volume of particles with C6 symmetry operation in 3D refinement and reconstruction would wash out the low noise level induced by low electron dose while elevate protein signals at those attained resolutions. Therefore, higher symmetry in biological assembly for single particle may significantly reduce sensitivity to radiation damage.

By contrast, global damage to a 3D protein crystal is composed of all the beam-induced damages to macromolecules randomly scattered in the whole illuminated volume. High resolution diffraction signal loss is proportional to approximately square of total number of defected unit cells as in expression (3), which makes MicroED a very sensitive method to measure protein damage by electron radiation. For 2D membrane protein crystals, which is assembled by macromolecules orderly arranged in a lipid bilayer, site specific damage rate as a function of dose should be very similar to SPA according to similarity of specimen thickness. When the dimension of a 3D crystal parallel to electron beam is thinner than electron inelastic scattering mean free path, diffraction intensity decay rate for high resolution spots would be proportional to square of electron dose comparing to linear relation for single particle [Grant and Grigorieff 2015]. When the dimension of a 3D crystal parallel to electron beam is greater than electron inelastic scattering mean free path, the number of layers in crystal will become an important factor in diffraction intensity decay rate. By incorporating this parameter, now investigators may be able to estimate the ratio of radiation damaged macromolecules at high resolution in SPA using series of exposures 3D crystal EDP with extremely low dose increment. The correlation is now expressed as:

$$N_{SPD}/N_{SP0}\;\propto \left[1-\sqrt{I_{d}/I_{o}}\right]/{\text{N}}_{c}$$ (4)

N_SPD_ and N_SP0_ represent radiation damaged and total selected macromolecule numbers respectively in single particle sample, I_0_ is intensity of a high resolution spot measured from first EDP in series exposure, I_d_ is the intensity from the same diffraction spot after accumulated certain dose, N_c_ is number of layers in crystal parallel to electron beam. Expression (4) explains that site specific damage or inelastic and elastic scattering ratios in single particle and 3D protein crystal are in the same magnitude at high resolution when crystal layers parameter is taken in consideration. Therefore, radiation damage results assessed by MicroED could be applied to estimate ratio of radiation damaged and total selected macromolecules at atomic resolution for SPA.

SPA, 3D electron crystallography, and tomography (cryo-ET) are the three powerful applications in structural biology using cryo electron microscopes. SPA and 3D electron crystallography can routinely determine macromolecule structure at atomic resolution, whereas more methods are in development to advance cryo-ET resolution envelope for cellular biology to near atomic resolution such as sub-tomogram averaging [Bouvette et al., 2021, Schur et al., 2014 and Zhao et al., 2013]. Data collection schemes and total electron dose needed per exposure vary greatly in these methods, as well as the distribution of accumulated radiation damages to sample in datasets. A MicroED dataset is consisted of series electron diffraction patterns recorded from continuously tilting 3D protein crystals at < 0.05 e^−^/Å^2^ per frame, until high resolution diffraction spots in a diffraction pattern have substantial loss. Diffraction patterns with high dose accumulation in a dataset would be discarded from 3D reconstruction [Hattne et al., 2018; Martynowycz et al., 2021]. Since a dataset records diffraction from a single specimen volume, removing later frames could remove radiation damaged signals but also reducing the data redundancy and structure completeness. To overcome this problem, more datasets from crystals in different orientations can be merged to reconstruct the final structures [Nannenga and Gonen 2019].

Total dose for a typical SPA dose fractionated movie ranges from 30 e^−^/Å^2^ to 60 e^−^/Å^2^ (50 e^−^/Å^2^ is the most common for 200–500 kDa protein complex). Progressively accumulated radiation damages to particles are recorded in later frames of an image stack during exposure. A few algorisms can be applied during frame alignment to maximize attainable resolution. When electron dose is evenly fractionated into i.e. 50 frames, each frame contains very limited signal and contrast. By applying low pass filtering [Wang et al., 2014] or positive b-factor [Scheres, 2014] to each frame during correlation fitting, SNR is significantly improved for higher accuracy in motion correction. More importantly, applying dose weighing during motion correction is now the standard procedure to minimize as much artifact and radiation damage for each exposure area or micrograph. Frame alignment programs such as Unblur [Grant and Grigorieff 2015] and Motioncorr2 [Zheng et al., 2017] apply a constant b-factor to each frame for fail-proof correlation fitting and then an exposure filter prior to frame summation using a predetermined dose accumulation formulation as described in Grant et al., 2015. DE_process_frames program allows investigators to apply a scaling factor to the exposure filtering weight to compensate differences in radiation tolerance between biological specimens [Spear et al., 2015]. By using dose weighing, high resolution signals will mainly come from earlier undamaged frames while later damaged frames will only contribute low resolution signals for maintaining high SNR and contrast.

During 2D and 3D classification, any individual particle that does not carry high-resolution information will be discarded as only the “best of the best” or undamaged particles reached to the final 3D reconstruction. Table 2 listed cryo-EM SPA statistics data from several macromolecules with atomic resolution X-ray crystallography structure determined, which is an indication of high protein homogeneity. After cycles of 3D classification at high resolution, the percentage of discarded particles ranges from 16 to 79%, which it is fair to assume that the majority of discarded particles were mostly from damaged macromolecules by electron irradiation. Other factors that affect inelastic to elastic scattering ratio, e.g., radiation damage rate, in macromolecules are molecular composition and side-chain property, electron energy, and other sample quality related factors. Furthermore, protein complexes with higher symmetry applied during SPA reconstruction may tolerate higher electron dose due to its averaging power in computation. It will still need more systematic and in-depth studies to quantitatively assess radiation damage including how dose weighing factor can be adjusted accordingly with different types of protein complexes in order to determine optimal exposure time for SPA data collection and to minimize structure artefacts in a variety of samples [Kato et al., 2021].

**TABLE 2:** Single particle data processing statistics.

	Apoferritin^a^	Apoferritin^b^	GABA_A_-β3^a^	Aldolase^b^	Ribosome^c^
Symmetry	O	O	C5	D2	C1
Subunits	24	24	5	4	1
Initial particles number	428,590	405,106	1,105,069	1,801,738	874,943
Final particles number	363,126	241,878	233,567	394,294	307,495
Ratio of final and initial	0.84	0.56	0.21	0.21	0.35
Acceleration voltage kV	300	200	300	200	300
Total dose e^−^/Å^2^	40	58	40	67	39.9
Resolution Å	1.22	1.75	1.73	2.13	2.02

a – Nakane et al., 2020.

b – Wu et al., 2020.

c – Watson et al., 2020.

Two microscope setting-related factors can also play a role affecting radiation damage rate. Dose rate might affect radiation damage rate to frozen-hydrated sample [Chen et al., 2008]. Using extremely low dose rate like in MicorED could allow much longer relaxation time for some ionized sites to be recovered. MicroED could be used to evaluate diffraction intensity decay dependence on dose rate at a very fine sampling increment, which may be able to capture more systematic information about this correlation. The acceleration voltage of electron beam also can affect radiation damage rate to protein molecules because inelastic scattering cross-section is reversely-proportional to electron energy [Peet et al., 2019]. Theoretically, in Table 2, cryo-EM single particle data collected by 300 kV microscopes should have lower radiation damage ratio than those collected by 200 kV microscopes with the same total dose.

In comparison to SPA and MicroED, a typical cryo-ET dataset consists of a series of electron exposures recorded as tilting sample from 0⁰ to ±60⁰ symmetrically with total dose ranged from 80 to 120 e^−^/Å^2^, and each micrograph corresponds to a sequentially discrete tilt angle at 1–3⁰ increment [Hagen et al., 2017]. Unlike SPA, cryo-ET distributes the accumulated dose throughout exposures at each tilt. Therefore, each exposure receives very low electron dose, hence very low SNR is observed in each tilt or projection. Using dose symmetric scheme, the low tilted exposures will have less electron dose accumulation with less effect from inelastic scattering due to low specimen thickness hence retaining more high resolution signal. By contrast, high tilted exposures will contain more severe radiation damage due to a combination of high dose accumulation in later exposures and increasing thickness drastically impacted by inelastic scattering effect. As a consequence, only low resolution signals could be used in 3D reconstruction resulting a large missing wedge in high resolution 3D reconstruction. Similar to MicroED, structure completeness can also be improved by merging multiple datasets, in this case tilt series, to record high resolution signals covering all angular orientations. Today, it remains unclear how much the radiation damage could reduce resolution limit for cryo-ET as there are still too many unidentified factors. Future systematic experiments that measure radiation damage in macromolecules by cryo-EM SPA and MicroED soon will provide more insights on resolution limit for cryo-ET and other newly developed methods.
